# Phosphate overload via the type III Na-dependent Pi transporter represses aortic wall elastic fiber formation

**DOI:** 10.20407/fmj.2023-004

**Published:** 2024-08-28

**Authors:** Yasumasa Yoshino, Tomoka Hasegawa, Shukei Sugita, Eisuke Tomatsu, Naoya Murao, Izumi Hiratsuka, Sahoko Sekiguchi-Ueda, Megumi Shibata, Takeo Matsumoto, Norio Amizuka, Yusuke Seino, Takeshi Takayanagi, Yoshihisa Sugimura, Atsushi Suzuki

**Affiliations:** 1 Department of Endocrinology, Diabetes and Metabolism, Fujita Health University, School of Medicine, Toyoake, Aichi, Japan; 2 Department of Developmental Biology of Hard Tissue, Division of Oral Health Science, Graduate School of Dental Medicine, Hokkaido University, Sapporo, Hokkaido, Japan; 3 Biomechanics Laboratory, Nagoya Institute of Technology, Nagoya, Aichi, Japan; 4 Department of Mechanical Systems and Engineering, Graduate School of Engineering, Nagoya University, Nagoya, Aichi, Japan

**Keywords:** Phosphate, Aorta, Elastin, Fibrillin, Atherosclerosis

## Abstract

**Objectives:**

Phosphate (Pi) induces differentiation of arterial smooth muscle cells to the osteoblastic phenotype by inducing the type III Na-dependent Pi transporter Pit-1/solute carrier family member 1. This induction can contribute to arterial calcification, but precisely how Pi stress acts on the vascular wall remains unclear. We investigated the role of extracellular Pi in inducing microstructural changes in the arterial wall.

**Methods:**

Aortae of Pit-1-overexpressing transgenic (TG) rats and their wild-type (WT) littermates were obtained at 8 weeks after birth. The thoracic descending aorta from WT and TG rats was used for the measurement of wall thickness and uniaxial tensile testing. Structural and ultrastructural analyses were performed using light microscopy and transmission electron microscopy. Gene expression of connective tissue components in the aorta was quantified by quantitative real-time polymerase chain reaction.

**Results:**

Aortic wall thickness in TG rats was the same as that in WT rats. Uniaxial tensile testing showed that the circumferential breaking stress in TG rats was significantly lower than that in WT rats (*p*<0.05), although the longitudinal breaking stress, breaking strain, and elastic moduli in both directions in TG rats were unchanged. Transmission electron microscopy analysis of the aorta from TG rats showed damaged formation of elastic fibers in the aortic wall. Fibrillin-1 gene expression levels in the aorta were significantly lower in TG rats than in WT rats (*p*<0.05).

**Conclusions:**

Pi overload acting via the arterial wall Pit-1 transporter weakens circumferential strength by causing elastic fiber malformation, probably via decreased fibrillin-1 expression.

## Introduction

Cardiovascular disease (CVD), including ischemic heart disease, atherosclerosis, stroke, and heart failure, are major concerns, especially in older people and patients with lifestyle-related diseases, such as diabetes, chronic kidney disease, and hypertension.^1^ Among them, aortic diseases consist of aneurysms, dissection, and occlusion resulting from atherosclerosis. The aorta, which is the blood vessel responsible for delivering blood from the heart to the systemic circulation, normally possesses a high degree of elasticity.^2^ The connective fibers within the microstructure of the aorta consist of elastin and collagen, which impart elastic properties and strength, respectively, to the aorta. Changes in the quantity and/or architecture of these fibers often lead to the mechanical and functional changes associated with aortic disease. Arteries show anisotropic and hyperelastic stress versus strain curves where the majority of the passive mechanical behavior is due to the extracellular matrix, which contains collagen and elastin.^3,4^ Fragmentation of elastin occurs with aging^5^ and at atherosclerotic lesions with calcification in the aorta,^6^ and the content of elastin is also decreased in the aorta in Marfan’s syndrome, which is often accompanied by aortic aneurysm.^7^ At calcified lesions in atherosclerosis, trans-differentiation of aortic smooth muscle cells (ASMCs) to the osteoblastic phenotype can occur, but degradation of elastin might precede osteoblastic differentiation of ASMCs.^8^ However, the precise mechanism leading to reduced arterial strength with aging and atherosclerosis has not yet been fully clarified.

Inorganic phosphate (Pi) in extracellular fluid plays important roles in maintaining cellular function.^9^ Pi uptake at the cellular membrane is essential for maintenance of cell viability through ATP synthesis, but Pi overload from the extracellular milieu causes cell stress.^10^ We have previously reported that high Pi medium induces cell death in rat A-10 vascular smooth muscle cells (VSMCs).^11^ Hyperphosphatemia is often encountered in chronic kidney disease-mineral and bone disorder (CKD-MBD), and the regulation of serum Pi concentrations plays critical roles in the development and progression of CKD-MBD.^12^ Hyperphosphatemia in patients with CKD is also associated with vascular calcification, particularly medial arterial calcification (Monckeberg’s medial sclerosis).^13^ Controlling serum Pi concentrations is vital for managing CKD-MBD and reducing the associated complications.

Pi influx from the extracellular milieu is controlled by an active Pi transport system driven by an inwardly-directed sodium (Na) gradient. There are at least three types of Na-dependent Pi transporters called types I, II, and III in mammalian cells.^9^ Among them, the type III Pi transport systems, namely Pit-1/solute carrier family 20 member 1 (Slc20a1) and Pit-2/Slc20a2, are ubiquitously expressed, and are considered essential for Pi influx in systemic cells. With regard to bone-forming cells, Pit-1 is also necessary for the induction of extracellular mineralization by expressing Pit-1 in matrix vesicles, which are released from osteoblasts and chondrocytes.^14–16^ However, low expression of Pit-1 in a mouse model showed normal bone mineralization with increased expression of Pit-2.^17^ Pit-2 is a causative gene for the brain arteriolar calcification in people with familial basal ganglion calcification.^18^ Bone-specific loss of Pit-2 shows abnormal bone development and decreased bone mineral density.^19^ Pit-2 may be a physiological regulator of tissue mineralization and may play critical roles in the determination of bone quality and strength.^20^ In addition to bone formation, arterial calcification is modulated by the transition of ASMCs from contractile to chondro-osseous forms. This program of trans-differentiation is thought to be induced through the expression of Pit-1. Pi uptake via Pit-1 is required for osteochondrogenic phenotypic changes and calcification of VSMCs *in vitro*.^21,22^ VSMCs isolated from targeted deletion of Pit-1 in VSMCs show the same aortic calcification as that with increased Pit-2 expression, and overexpression of Pit-2 restores Pi uptake and Pi-induced calcification in Pit-1-deficient VSMCs.^23^ These findings suggest a compensatory role of Pit-2 for Pit-1. In contrast, Pit-2 in VSMCs protects against Pi-induced vascular calcification in mice with CKD.^24^ These findings suggest that Pit-1 and Pit-2 play major roles in physiological and pathological mineralization, and serve redundant roles in Pi-induced calcification of VSMCs.^23,24^ We have previously shown that overexpression of Pit-1/Slc20a1 in rats reduces their life-span^25^ and nephrotic syndrome (progressive proteinuria and hypoalbuminemia) results from Pi-induced glomerular podocyte injury in their kidney.^26^ These rats also show impaired incisor formation and low bone mass with a normal length of their extremities.^27,28^ These findings suggest that chronic Pi overload in each organ could damage their function in Pit-1-overexpressing transgenic (TG) rats.

In the present study, we examined the mechanical strength of the aortic wall in TG rats overexpressing the type III Pi transporter, Pit-1/Slc20a1, to examine the effect of Pi “stress” on the aortic wall *in vivo*.

## Methods

### Animals

The mouse Pit-1/Slc20a1 gene was ubiquitously expressed under the control of a cytomegalovirus early enhancer element and chicken β-actin (CAG) promoter in TG rats after pronuclear DNA microinjection into rat zygotes as previously described.^26,27,29^ All rats were housed at 24°C with a 12-h/12-h light/dark cycle and were allowed free access to tap water and a normal rodent chow. Generally, TG rats died at 24–32 weeks because of malnutrition and cachexia, while wild-type (WT) rats generally survived more than 2 years.^25^ Routine serum chemistries were measured using a Hitachi 7180 automatic analyzer (Hitachi High Technologies, Tokyo, Japan). All of the rats were anesthetized with ether and then killed by exsanguination from the abdominal aorta.

This research adheres to the Guiding Principles for the Care and Use of Animals in Research adopted by the American Physiological Society. All animal procedures and a statement of protocol were approved by the institutional animal care and use committee (#M0201, approved on 1 April 2007) and the relevant government authorities.

### Light microscopy

Thoracic descending aortae of TG (n=3) and WT (n=3) rats were fixed in 4% paraformaldehyde solution diluted in 0.1 M phosphate buffer (pH 7.4) for 24 h. The fixed samples were rinsed by phosphate-buffered saline and then dehydrated in ascending ethanol solutions prior to paraffin embedding. Histological sections of 5-μm thickness were prepared in the horizontal direction of the thoracic descending aorta. They were deparafinized and rehydrated, and then subjected to hematoxylin–eosin staining or van Gieson staining, which is a simple method of differential staining of collagen and other connective tissues including elastic fibers. In van Gieson staining, the sections were stained with Weigert’s resorcin-fuchsin solution for 40 min, Weigert’s iron hematoxylin for 30 min, and van Gieson solution for 5 min at room temperature. The lumen area of the thoracic aorta in horizontal paraffin sections with hematoxylin-eosin staining and elastic fiber/elastic plate area in the aorta in those with van Gieson staining were measured by Image-Pro Plus 6.2 software (Media Cybermetrics, Inc., Bethesda, MD, USA).

### Electron microscopy

Thoracic descending aortae of TG (n=3) and WT (n=3) were fixed in a mixture containing 2.5% glutaraldehyde and 2% paraformaldehyde in 0.067 M phosphate buffer (pH 7.4) for 18 h. After washing with phosphate-buffered saline, the samples were post-fixed in 1% OsO_4_ for 4 h, dehydrated with ascending concentrations of acetone, and then embedded in epoxy resin (Taab Laboratories Equipment Ltd., Aldermaston, UK). Ultrathin sections of 120-nm thickness were obtained using an ultramicrotome (Sorvall MT-5000; Ivan Sorvall, Inc., Norwalk, CT, USA). The ultrathin sections were stained with uranyl acetate and lead citrate prior to observation by transmission electron microscopy (Hitachi H-7100; Hitachi Co. Ltd, Tokyo, Japan) at an accelerating voltage of 80 kV.

### Measurement of mechanical strength of the aortic wall

Thoracic descending aortae of TG (n=6) and WT (n=6) rats were obtained at 8 weeks after birth and used for measurement of wall thickness and for uniaxial tensile testing. Tubular segments of 18 mm in unloaded length were obtained from the first branch of the intercostal arteries and divided into halves. Each of the proximal and distal halves was then cut longitudinally and opened flat. A dumbbell-shaped specimen was cut out for tensile testing in the circumferential and longitudinal directions, which was performed in saline solution at room temperature at a rate of 5 mm/min. Stress was calculated as nominal stress (i.e., force/[initial thickness×initial width]). Strain was calculated as follows: (length–initial length)/initial length. Stress–strain curves were fitted with two straight lines using the Solver function of Excel (Microsoft Corp., Redmond, WA, USA). Slopes of the straight lines in low- and high-strain regions were calculated as indices of elastic moduli of elastin *E*_e_ and collagen *E*_c_, respectively. Strain at the intersection of the two straight lines was obtained as an index of critical strain *ε*_crit_ at which the major load-bearing component changes from elastin to collagen.

### Quantitative real-time polymerase chain reaction

Total RNA was isolated from the thoracic descending aortic wall of TG (n=6) and WT (n=6) rats using RNeasy spin columns (Qiagen, Hilden, Germany), and cDNA was synthesized using the SuperScript III first strand synthesis system (Thermo Fisher Scientific, Waltham, MA, USA). Quantitative real-time polymerase chain reaction (qRT-PCR) was performed using the TaqMan probes described below (Thermo Fisher Scientific) and TaqMan Gene Expression Master Mix using the ABI PRISM7900HT Sequence Detection System (Applied Biosystems, Foster City, CA, USA). Each amplification was compared with amplification of the house-keeping gene GAPDH. The assay IDs of TaqMan probes used for qRT-PCR were as follows: rat collagen type 1 (*Col1a1*), Rn01463848_m1; rat elastin (*Eln*), Rn01499782_m1; rat fibrillin-1 (*Fbn1*), Rn01514895_m1; rat fibrillin-2 (*Fbn2*), Rn00582832_m1; rat matrix Gla protein (*MGP*), Rn00563463_m1; rat Runt-related transcription factor 2 (*Runx-2*), Rn01512298_m1; and rat *GAPDH*, Rn01775763_g1. PCR was performed at 95°C for 15 s and 60°C for 60 s (40 cycles). To quantify *Slc20a1* and *Slc20a2*, cDNA was synthesized using the CellAmp Direct Lysis and RT set (Cat# 3737S/A; Takara Bio, Shiga, Japan) according to the manufacturer’s instructions. qRT-PCR was performed using the QuantStudio 7 Flex system (RRID:SCR_020245; Thermo Fisher Scientific) using TaqMan Universal Master Mix II with UNG (Cat# 4440038; Thermo Fisher Scientific). Each amplification was compared with amplification of the house-keeping gene *Actb*. The assay IDs of TaqMan probes used for qRT-PCR were as follows: rat *Actb*, Rn00667869_m1; rat *Slc20a1*, Rn00579811_m1; and rat *Slc20a2*, Rn00568130_m1. The primer used for *Slc20a1* recognizes mouse and rat Pit-1. PCR was performed at 95°C for 15 s and 60°C for 60 s (50 cycles).

### Statistical analysis

The results are expressed as the mean±standard deviation. The two-tailed unpaired Student’s *t*-test was used for statistical analysis. Differences between the experimental groups were considered significant when the *p* value was <5%.

## Results

### Macroscopic findings of the thoracic descending aorta in TG and WT rats

**Figure 1:**
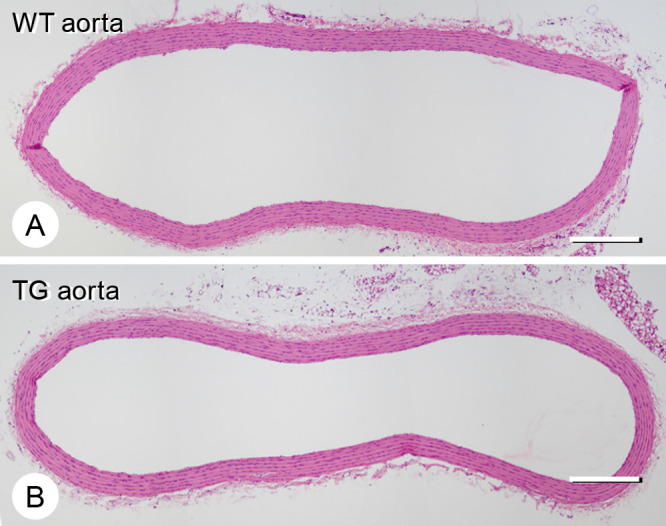
Hematoxylin–eosin staining of wild-type (WT) and type III phosphate transporter transgenic (TG) thoracic descending aortae. The thickness of the aortic wall in TG rats (B) appeared the same as that in WT rats (A). There were three rats in each group. Representative figures are shown. Bars, 400 μm.

No macroscopic atherosclerosis or ectopic calcification was observed in the thoracic descending aorta in TG or WT rats (Figure 1). The lumen area and elastic tissue/aorta wall area in TG rats (n=3) were identical to those in WT rats (n=3) (lumen area: WT, 1.31±0.3 mm^2^; TG, 1.28±0.2 mm^2^ and elastic tissue area/aortic wall: WT, 48.4%±1.1%; TG, 45.3%±5.0%).

### Measurement of mechanical strength of the thoracic descending aortic wall in TG and WT rats

**Figure 2:**
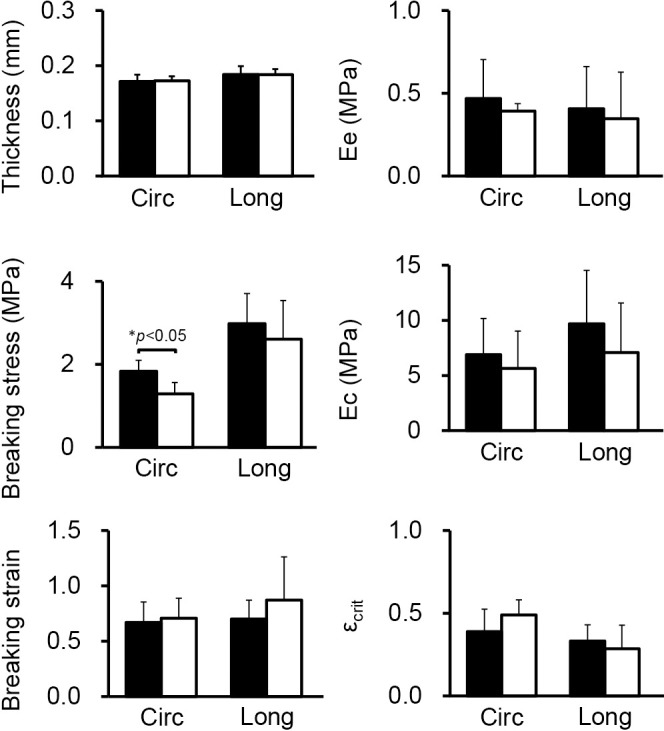
Thoracic descending aortae of type III phosphate transporter transgenic rats (open bar) (n=6) and their wild-type littermates (closed bar) (n=6) at 8 weeks old were used for measurement of wall thickness and uniaxial tensile testing. The unloaded wall thickness, breaking stress and strain, indices of elastic moduli of elastin *E*_e_, and collagen *E*_c_, and an index of critical strain *ε*_crit_ at which the major load-bearing component is changed from elastin to collagen were obtained for circumferential (Circ) and longitudinal (Long) directions. *p<0.05 was considered significant.

The thickness of the thoracic descending aortic wall in TG rats was the same as that in WT rats (Figure 2). However, uniaxial tensile testing showed that the circumferential breaking stress in TG rats was significantly lower than that in WT rats (*p*<0.05). The longitudinal breaking stress, breaking strain, elastic moduli, and critical strain in both directions in TG rats were the same as those in WT rats (Figure 2).

### Histological and ultrastructural analysis of elastic fibers of the thoracic descending aorta in TG and WT rats

**Figure 3:**
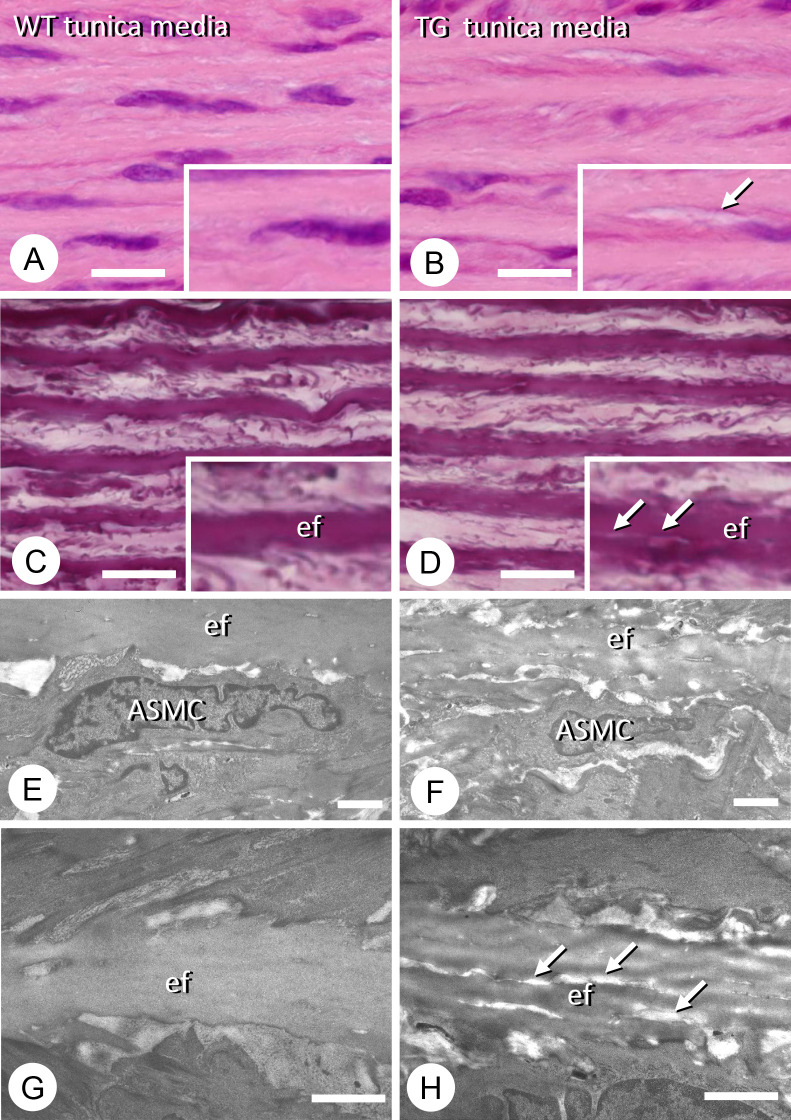
Histological and ultrastructural observations of the thoracic descending aortae of wild-type (WT) and type III phosphate transporter transgenic (TG) rats. There were three rats in each group. Representative figures are shown. Hematoxylin–eosin staining of the tunica media of the descending aorta in WT rats (A) and in Pit-1 TG rats (B). There appears to be some fissures in the elastic fibers (ef) of the tunica media in the TG aorta (see arrow in inset, B) compared with the WT counterparts (A). van Gieson staining shows the presence of small tears in the elastic fibers of the TG tunica media (D), but not in the WT elastic fibers (C). The arrows indicate tears in an elastic fiber in the inset of D, while WT elastic fibers show even van Gieson staining (inset of C). Transmission electron microscopy images of arterial smooth muscle cells (ASMCs) and elastic fibers (ef) in the tunica media of WT (E) and TG (F) rats. When observed at a higher magnification, the WT elastic fibers show uniform electron density (G), while the TG elastic fibers contain fine fissures (arrows, H). Bars, A–D: 10 μm; E, F: 2 μm; G, H: 1 μm.

Histologically, there were many fissures, which were not found in WT, within the elastic fibers of the thoracic descending aorta in all TG rats examined, although smooth muscle cells appeared intact (Figure 3A–D). A ultrastructural analysis of the thoracic descending aorta in TG rats by transmission electron microscopy showed damaged formation of elastic fibers in the aortic wall (Figure 3E–H).

### Gene expression by qRT-PCR of the thoracic descending aorta in TG and WT rats

**Figure 4:**
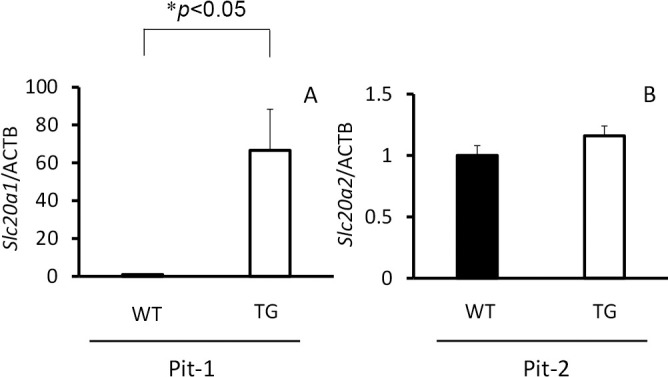
*Slc20a1* and *Slc20a2* gene expression in thoracic descending aortae of type III phosphate transporter transgenic (TG) rats (n=6) (open bar) and their wild-type (WT) littermates (n=6) (closed bar) at 8 weeks old. (A) Pit-1 (*Slc20a1*) and (B) Pit-2 (*Slc20a2*). *p<0.05 was considered significant.

**Figure 5:**
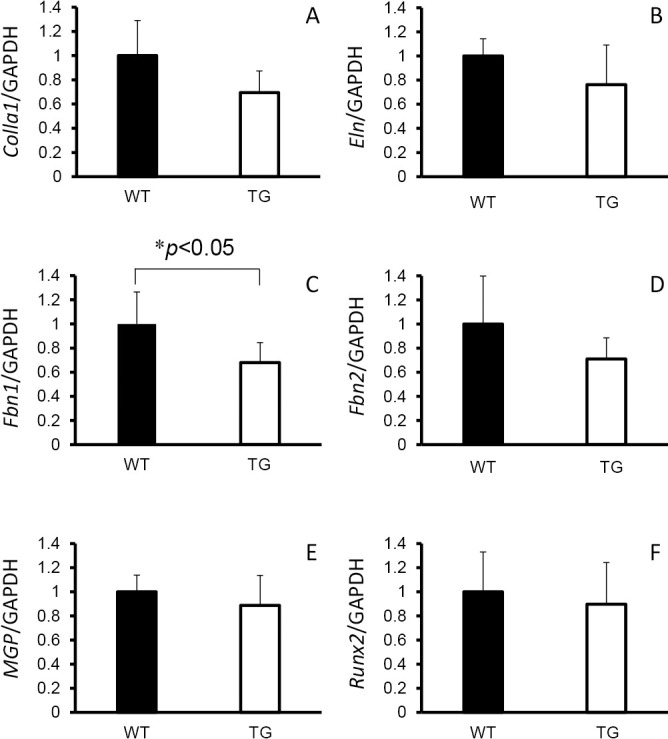
*Col1a1*, *Eln*, *Fbn1*, *Fbn2*, *MGP*, and *Runx-2* gene expression in thoracic descending aortae of type III phosphate transporter transgenic (TG) rats (n=6) (open bar) and their wild-type littermates (WT) (n=6) (closed bar) at 8 weeks old. (A) *Col1a1*, (B) *Eln*, (C) *Fbn1*, (D) *Fbn2*, (E) *MGP*, and (F) *Runx-2*). *p<0.05 was considered significant.

With regard to Pi transporter-related genes in the thoracic descending aortae, there was a significant increase in *Slc20a1* (Pit-1) expression in TG rats (*p*<0.05), but there was no increase in *Slc20a2* (Pit-2) expression (Figure 4). Elastic fiber-related gene expression in the thoracic descending aorta showed a significant decrease in *Fbn1* expression in TG rats (*p*<0.05), but there was no change in expression of *Col1a1*, *Eln*, *Fbn2*, *MGP*, or *Runx2* (Figure 5).

## Discussion

In the present study, we found that overexpression of the type III Na-dependent Pi transporter Pit-1/Slc20a1 damaged formation of the elastic fiber component of the thoracic descending aorta in rats, causing weakness against circumferential force. Collagen and elastin fibers are the two major components of the aortic wall, and they contribute to resistance to the mechanical stress exerted by blood pressure.^2^ In our animal model, Pi transporter-overexpressing TG rats showed a significant decrease in gene expression of *Fbn1*, which form elastic fibers.

Pi plays a critical role in arterial calcification and in the prognosis of CKD-MBD.^12^ In the uremic status with end-stage renal disease, an elevation in serum Pi concentrations is related to arterial calcification partly through trans-differentiation of ASMCs to osteoblastic cells in arterial walls.^21,22^ Kuro-o^30^ also reported that Pi plays a major role in the processes of aging and arterial calcification. In our Pit-1-overexpressing rats, the Pi transport activity of primary cultured ASMCs was two-fold higher than that in WT rats, but ectopic calcification in the aortic wall was not apparent.^25^ Pit-1 and Pit-2 serve redundant roles in Pi-induced calcification of VSMCs,^23^ and Pit-2 in VSMCs could protect against Pi-induced vascular calcification.^24^ In fact, TG rats did not always show arterial calcification before their death at 8 months of age. This finding suggests that Pi overload alone may not induce arterial calcification *in vivo*.

However, some TG rats developed thin and fragile aortae compared with WT rats. As we previously reported, aged Pit-1 TG rats have massive proteinuria with hypoalbuminemia,^26^ and the damage to the aortic wall of TG rats might be due to systemic loss of protein. Therefore, we examined structural changes in the aortic wall at 8 weeks of age when TG rats do not show severe hypoalbuminemia or massive proteinuria. In general, there were no differences in the wall thickness or shape of the wall between TG and WT rats at 8 weeks old. However, we found that the circumferential breaking stress of the thoracic descending aorta in TG rats was significantly lower than that in WT rats, suggesting Pi-induced structural changes in the aortic wall in these rats. The mechanism of extracellular Pi on cellular function and the Pi-sensing mechanism are not fully understood. Several studies have indicated the involvement of Pit-1 and Pit-2 in the process of Pi sensing.^31^ Pit-1 and Pit-2 mediate Pi uptake in mammalian cells. Additionally, extracellular Pi upregulates Pit-1 and Pit-2 expression, which involves the extracellular signal-regulated kinase 1/2 signaling pathway.^32^ Pi-induced apoptosis of chondrocytes is also dependent upon the activation of this signaling pathway.^33^ However, Pi binding rather than Pi uptake can contribute to Pi signaling through Pi transporters,^34^ and Pi uptake-dependent and Pi uptake-independent function of Pit-1 in VSMCs appears to be important for vascular calcification.^35^ Further investigation on this issue is required.

An ultrastructural analysis of the aorta in TG rats showed damaged formation of elastic fibers accompanied by reduced *Fbn1* expression. Elastin plays an important role in elasticity of the aortic wall but the total elastin content does not change with aging.^2^ However, fragmentation of elastin occurs along with remodeling of the aorta.^5^ This fragmentation has been reported to be present in calcified thoracic aortae with atherosclerotic lesions.^36^ In addition, elastin degradation might precede osteoblastic differentiation of ASMCs.^8^ These findings suggest that chronic Pi overload could accelerate, at least in part, the aging process of the aortic wall.

Elastic fibers consist of an inner core of elastin and a peripheral mantle of fibrillin-rich microfibrils.^36,37^ Fibrillins are extracellular glycoproteins, and they provide mechanical support in the form of microfibrils along with elastin. The fibrillin superfamily comprises three isoforms of fibrillin-1, -2 and -3. Fibrillin-1 is the main component of microfibrils and provides the scaffold for elastin deposition during the formation of elastic fibers.^38^ Mutation of the *Fbn1*gene causes Marfan’s syndrome, and cardiovascular complications including aortic aneurysm are the major cause of death in this syndrome.^39^ Fibrillin-1 and fibrillin-2 have differing functional roles. Fibrillin-2 may possess a major functional role during early morphogenesis in directing elastic fiber assembly.^40^ In contrast, the predominance of fibrillin-1 in stress- and load-bearing structures suggests that fibrillin-1 may be mainly responsible for the structural function of microfibrils.^41^ In this study, we showed that Pit-1 overexpression induced deformity of elastic fibers together with decreased *Fbn1*gene expression. Although the effect of Pi with overexpression of Pit-1 did not affect the total amount of elastic fibers, Pit-1 overexpression might have damaged the development of elastic fibers through a decrease in fibrillin-1 in the aortic wall, resulting in structural weakening.

There are several limitations to this study. First, the Pi transporter overexpression in this animal model was ubiquitous and not specific to the aortic wall, and there was a lack of data of the role of another Pi transporter, Pit-2/Scl20a2. Second, Pi transporter overexpression in this animal model occurred since birth and does not fit the development of hyperphosphatemia in adult patients with end-stage renal disease. Third, although the excess of Pi uptake by overexpression of Pi transporters can induce cellular stress by several pathways such as extracellular signal-regulated kinase 1/2, we could not determine the precise mechanism of reduced fibrillin-1 formation. Fourth, the direct effect of dietary Pi on the progress of aortic wall damage due to Pi transporter overexpression throughout life is unclear because nephrotic syndrome was severe in this animal model. We previously showed that high Pi diet-treated TG rats showed more progressive proteinuria and higher serum creatinine concentrations than normal Pi diet-treated TG rats.^26^

In conclusion, Pi overload, acting via the Pit-1 transporter in the arterial wall, weakens the circumferential strength of the vessel as a result of malformation of elastic fibers with a decrease in fibrillin-1.
